# Healthcare4VideoStorm: Making Smart Decisions Based on Storm Metrics

**DOI:** 10.3390/s16040588

**Published:** 2016-04-23

**Authors:** Weishan Zhang, Pengcheng Duan, Xiufeng Chen, Qinghua Lu

**Affiliations:** 1 Department of Software Engineering, China University of Petroleum, No. 66 Changjiang West Road, Qingdao 266031, China; chaseandblack@gmail.com (P.D.); dr.qinghua.lu@gmail.com (Q.L.); 2 Hisense TransTech Co., Ltd., No. 16 Shandong Road, Qingdao 266031, China

**Keywords:** storm metrics, CPU-GPU, scheduling, optimization

## Abstract

Storm-based stream processing is widely used for real-time large-scale distributed processing. Knowing the run-time status and ensuring performance is critical to providing expected dependability for some applications, e.g., continuous video processing for security surveillance. The existing scheduling strategies’ granularity is too coarse to have good performance, and mainly considers network resources without computing resources while scheduling. In this paper, we propose Healthcare4Storm, a framework that finds Storm insights based on Storm metrics to gain knowledge from the health status of an application, finally ending up with smart scheduling decisions. It takes into account both network and computing resources and conducts scheduling at a fine-grained level using tuples instead of topologies. The comprehensive evaluation shows that the proposed framework has good performance and can improve the dependability of the Storm-based applications.

## 1. Introduction

Large scales of data, especially streaming data, are accumulating everyday through the usage of widely-deployed video cameras, mobile devices and social networks, like Twitter and Facebook. As the biggest Big Data [1], surveillance video has become an important source for mining valuable information. Real-time performance is important for many smart city applications, such as automatic traffic jam detection, crime detection and criminal tracking. These kinds of applications require powerful computing resources and can adopt Storm for processing video streams. In order to provide high dependability for such applications, it is necessary to utilize all-possible computing resources, *i.e.*, both the CPU and GPU at their best. Additionally, knowing the status of running tasks (especially detecting performance bottlenecks) and scheduling resources to make an application run smoothly are critical to making stream processing-based applications dependable.

There are efforts to enhance Storm’s capabilities. For example, Lutz *et al.* [2] presents NaaStorm, which is an implementation of Storm modelled on NaaS (Network as a Service), which can make the network infrastructure more transparent to tenants. It uses a round-robin mechanism from Storm to evenly distribute the execution of topology components on available machines, without considering the network cost when tuples go through the whole topology. Aniello *et al.* [3] propose a new scheduler for Storm to monitor system performance and reschedule the deployment at run-time. Fischer *et al.* [4] and Xu *et al.* [5] also propose a new scheduler that can detect network traffic and reschedule a topology execution. These works are dedicated to the topology scheduling to achieve resource optimization. However, scheduling a topology in Storm costs 5 to 30 s in our experiments, which, in the context of real-time analysis of video data in large scales, might be a disaster because some applications, such as automatic traffic jam detection and run-time detection of suspects from surveillance videos, are time critical. Further, these works only consider optimization of network resources and fail at taking computing resources into account. The analyses of big video data are both data intensive and computation intensive. Optimization of computing resources is very important for the assurance of QoS (Quality of Service) in real-time analysis of big video data.

Therefore, in this paper, we propose Healthcare4VideoStorm, a framework that helps obtain insights into Storm running status. It senses each video task running on Storm and extracts metrics from those tasks. Healthcare4VideoStorm consists of several components, including a metric sensor, a bottleneck detector, a router and a CPU-GPU switcher. The metric sensor collects metrics from Storm and sends them to the monitor, based on which the remaining three components can obtain metric data to conduct their own functionalities. The bottleneck detector detects Storm task performance degradation. The router periodically retrieves the configuration of video tasks on Storm and tries to optimize the network load. At a fine-grained level, the router schedules tuples instead of topologies. The router can balance the network load in a Storm cloud to remove the bottleneck. The CPU-GPU switcher calculates the throughput of each video task and compares it to the required throughput. When the calculated throughput is detected to be less than the required throughput, it switches workloads from the CPU to the GPU to ensure the required performance.

Intelligent processing on large-scale video data is very computation intensive and data intensive, which can easily cause resource bottlenecks. The four components are collaborating to collect the running status of each video task based on which bottlenecks can be detected. If a Storm cloud is a person, then the framework can be regarded as an auxiliary device that can achieve monitoring, detection and problem-solving for the person to keep him or her in a healthy manner. This is the reason why we name the proposed framework Healthcare4VideoStorm. The contributions of this paper include:

- A dedicated framework for obtaining Storm run-time metrics and conducting optimized scheduling that makes full use of computing resources. The framework can help make sure that Storm-based applications run smoothly for continuous stream processing.
- A fine-grained task scheduling strategy is designed and implemented to improve performance. It considers both network and computing resources in order to get optimized scheduling decisions.
- We have comprehensively evaluated the proposed framework in terms of its capabilities to find performance bottlenecks and the performance of switching processing units. It shows that the proposed framework is effective and efficient.

The remainder of the paper is organized as follows: First, we give a brief introduction of Storm in Section 2. Then, we present the design and implementation the components in the proposed Healthcare4VideoStorm in Section 3 and Section 4 evaluates the proposed framework. Section 5 presents some related works. Conclusions and future work end the paper in Section 6.

## 2. Background on Storm and Metrics Definition

**Figure 1:**
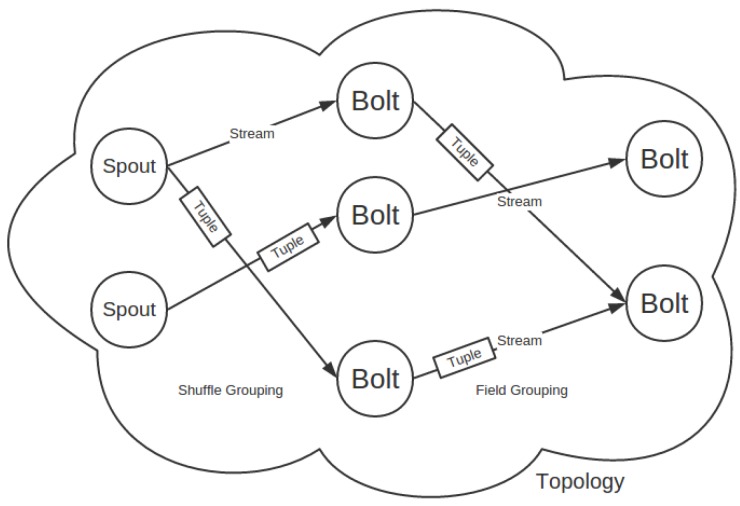
Storm topology.

A Storm platform can have several jobs called topologies. A topology mainly consists of spouts, bolts, tuples, streams and grouping specifications, as illustrated in Figure 1. Spout instances can get data and emit them into the rest of a topology. There are several methods to get data. One is to collect data directly from one device, which usually provides its APIs, such as Twitter’s Streaming API and Java OpenCV’s API. An alternative is to build a message queue as a middleware between devices and your topology. We choose this method in setting up the test environment. Another approach is the DRPC(Dirtributed Remote Procedure Call) call aimed at online processing. After being generated from spouts, tuples are emitted into bolts, which can consume the input tuples, carry out processing and possibly emit new streams. Streams bridge those bolts by groupings, which specify how tuples are emitted into the downstream bolts.

### 2.1. Parallel Mechanisms of Storm

A Storm cluster can hold several topologies. Each computer is configured with slots. Each topology is spawned by one or more workers (JVM (Java Virtual Machine) processes) allocated in different slots of different computers. Each worker usually offers several executors (Java threads). Each executor runs one or more tasks (one task in Storm’s default configurations). Those tasks, as a matter of fact, run component (spout and bolt) instances and are the computing cells that consume tuples. How the stream(s) is consumed is specified by Storm’s grouping modes, which can be shuffle grouping, field grouping, all grouping, direct grouping, global grouping and custom grouping. Which stream grouping should be used depends on the business logic residing in the bolts. One example can be a video target recognition application where multiple features (e.g., SURF and HOG) need extracting and combining. If we give a bolt to each feature, all grouping must be adopted, meaning that each video frame should be copied and emitted into each of those bolts. Another example lies in modelling video backgrounds. Multiple frame streams are pushed/pulled into a topology. In this case, field grouping should be adopted because modelling a background needs image frames from the same video stream.

### 2.2. Scheduling in Storm

Network transmission can be the bottleneck in a distributed system with high I/O throughput, for example, in the proposed framework, when the traffic among tasks located in different nodes puts a great challenge on the network. Here, we take a special focus on the task scheduling to minimize the network load. The default scheduler in Storm is called Even Scheduler, which adopts a round-robin fashion to produce an even allocation. A component (a spout or a bolt) may have one or more tasks. Once a topology is uploaded to a Storm cluster, all tasks of the component in that topology are assigned into one or more executors, which are assigned into one or more workers in the same way. Each worker is then assigned into an available slot.

**Figure 2:**
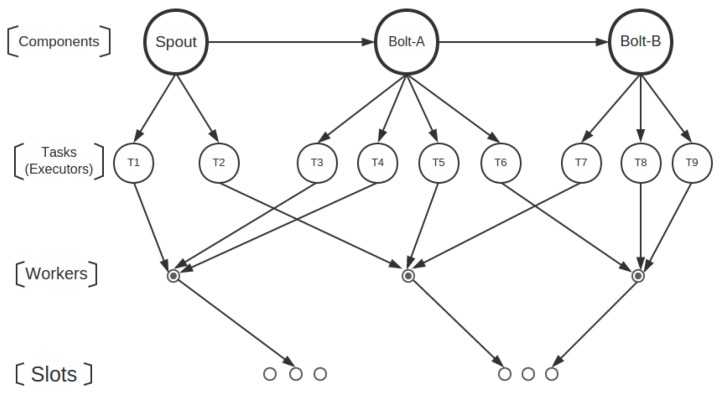
Storm scheduling.

Figure 2 illustrates a simulation of task scheduling where a three-component topology is running on a Storm cluster. The component spout generates tuples and emits them into the target downstream Bolt-A, where those tuples are consumed and again emitted into the next downstream component, Bolt-B, as each arrow between the components directs. Each component can be instanced into one or more tasks aligned with an ID. For example, Bolt-A is instanced into four tasks, which are bound with the IDs 3, 4, 5, 6, respectively. The number of tasks for each component is also called parallelism hint and is configured by developers.

In general, the scheduling process is triggered once a topology gets uploaded. The Storm scheduler scans cluster status and topology assignment, then decides which tasks can be assigned into the same worker and which worker is assigned to which slot based on the scheduling algorithm designed by developers. When the scheduling process assigns workers to slots, it gets involved with physical network transmission, which is what the scheduling algorithm must consider.

The Storm default even scheduling does not consider resource payload, especially the network. Leonardo *et al.* [3] propose an offline schedule that takes the component order into account and an improved online schedule that not only takes into account the component order, but also the real-time situations, like grouping specifications. The goal is to assign the tasks that follow the order and real-time groupings into one worker, thus reducing the network transmission.

In general, task scheduling that abides by both the component order and real-time grouping specifications is called online scheduling. It requires less network resources than task scheduling, which only considers the component order, which is called offline scheduling. The offline scheduling costs less than the default scheduling. For example in the default scheduling, one worker contains tasks T4, T5 and T6, while in the offline scheduling, one worker may contain tasks T1, T3 and T7. Because tuples begin with tasks of spout through that of Bolt-A and end in that of Bolt-B, tasks T4, T5 and T6 in the worker of the default scheduling do not communicate with each other, but perform tuple transmission with tasks of other workers. This surely increases the network traffic when compared to offline scheduling in which there exists a high likelihood that T1, T3 and T7 communicate with each other in the same worker where data transmission simply handles data pointers. However, it does not always happen, for it does not consider grouping specifications, yet online scheduling makes it more likely. A typical situation is that direct grouping is adopted between the three components where T2 emits tuples directly to T3 and T3 to T7. The online scheduling then assigns T2, T3, T7 into a worker instead of T2, T3, T7, thus further reducing the network traffic.

### 2.3. Metrics

Different topologies may focus on different metrics. For example, throughput is taken more into account than latency for stream processing topologies where batching techniques are used to increase throughput at the cost of worse latency. However, for distributed RPC topologies, latency is the key metric and usually optimized. Several topology-level metrics, which we consider critical, are:

- *Latency*: includes complete latency, execute latency and process latency. For complete latency, the timer is started when the tuple is emitted from the spout, and it is stopped when the tuple is acked. Therefore, it measures the time for the entire tuple tree to be completed. Process latency ends its timer when ackis called if we consider Storm’s ackingsystem.
- *Tuple staid*: snapshot the tuple population that stays in the task’s message queue.
- *Tuple throughput*: the tuple population that passes through the topology in the given time interval.
- *Traffic*: the data bulk that passes through the topology in the given time interval.
- *Ack count*: the number of tuples that have been successfully processed in one task.
- *Emit count*: the number of tuples that have been successfully emitted in one task.
- *CPU utility*: the ratio of one tuple’s CPU time fractions consumed in one task to its total time interval stayed in the task’s execute function.

## 3. Design and Implementation of Healthcare4VideoStorm

### 3.1. Architecture of Healthcare4VideoStorm

**Figure 3:**
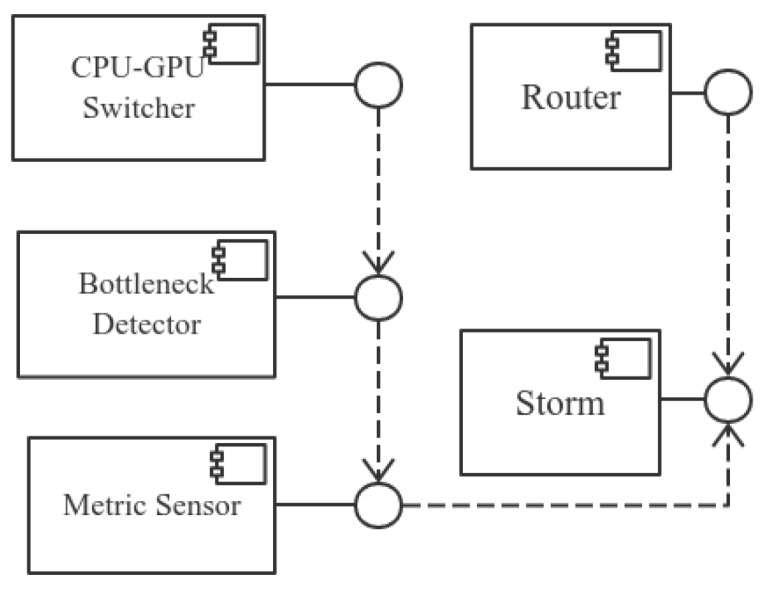
Healthcare4VideoStorm architecture illustrated as a component-connector view.

Before describing each component of the proposed framework, we give an overview of the architecture of Healthcare4VideoStorm, which is illustrated as a component-connector view (Figure 3). The metric sensor and the router directly depend on Storm. The metric sensor senses each task’s metrics by calling Storm metric interfaces; the router periodically calls Storm’s configuration so as to know the states of each task. The bottleneck detector reads the metrics obtained by the metric sensor and builds up a model to find abnormal patterns in these metrics. The CPU-GPU switcher is notified and then triggered by the bottleneck detector when it finds abnormal patterns.

### 3.2. Metric Sensor

The functionality of this component is to sense metrics from Storm tasks, to transmit and store them for further decision making. Storm provides Thrift and built-in metric interfaces for accessing topology-level and cluster-level metrics. Some metrics, such as traffic and CPU utility, however, cannot be obtained by Storm APIs. The metric sensor is designed to solve this problem.

**Figure 4:**
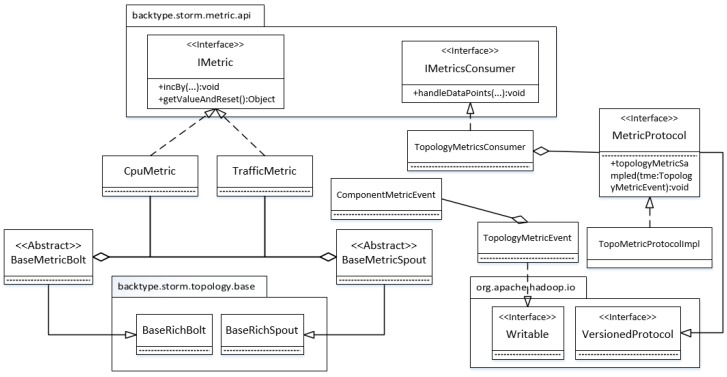
Metric sensor.

Figure 4 shows the implementation details of this component. A topology can be devised with a metric sensor through two steps. First, its spouts and bolts should implement BaseMetricSpout and BaseMetricBolt which extend the BaseRichBolt and BaseRichSpout of Storm respectively. Second, we need to set TopologyMetricsConsumer as the default metric consumer. Once the topology runs up, the consumer bolt will receive a metric message within a bucket time. The message is then wrapped into a TopologyMetricEvent object, which is sent to the monitor side through Hadoop RPC (Remote Procedure Call), which is used as the communication protocol for metric transmission. To make a TopologyMetricEvent object serializable, it must implement a Writable throughout interface. Because a topology consists of several components, a TopologyMetricEvent object that composes ComponentMetricEvent objects, should implement the Writable interface. Hadoop RPC also requires the implementation of the VersionedProtocol interface to pin down what to do with a sampled metric event.

### 3.3. Bottleneck Detector

**Figure 5:**
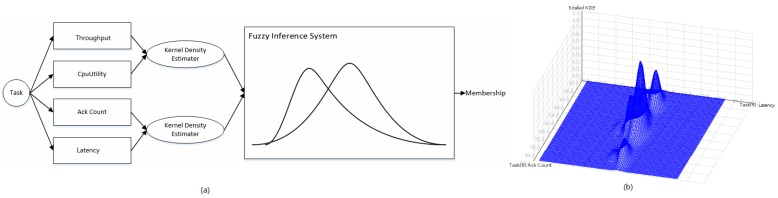
Bottleneck detector: (**a**) Fuzzy Inference System (**b**) Kernel Density Estimator.

Detecting bottlenecks in Storm is challenging in that the detector should operate in an online manner. It also requires that the detection algorithm should not be supervised and should be light-weight, yet accurate due to severe resource constraints. It is impractical to obtain training labels of a supervised model for each task of each topology in Storm; not only because it takes much hand-crafted labour, but also the label is hard to decide for different types of topologies in different application domains. Therefore, we adopt an unsupervised, light-weight approach to detect a Storm bottleneck, as shown in Figure 5a.

For each task, we extract four metrics, *i.e.*, latency, CPU utility, ack count and throughput, as detailed in Section 2.3. Every two metrics form a bivariate probability density distribution using the Gaussian Kernel Density Estimator (KDE). To shape a Storm task in this paper, one joint probability distribution is statistically built on latency and CPU utility, while the other joint probability distribution is built on ack count and throughput using KDE. In a certain initial state period of a task, metrics from the task are sampled in sequences, which are used to train the two probability distributions. Figure 5b shows the joint probability distribution of ack count and throughput using KDE. After the initial training, each newly-sampled metric will be fed into the two probability distributions. Probability estimations generated from the two probability distributions indicate the frequencies that metric values happen. These two estimated probabilities will be fed into a FIS (Fuzzy Inference System) [6], which uses three IF-ELSE rules to map inputs (the two estimated probabilities) to outputs (the membership/likelihood that the task runs in the degradation). This is detailed in Algorithm 1 using FCL (Fuzzy Control Language, which was standardized by IEC 61131-7 [7]).

**Algorithm 1** Fuzzy inference system.FUNCTION_BLOCK:  anomalydetection      TERM medium:=   gauss    0.5    0.12;VAR_INPUT      TERM high:=   (0.75, 0)    (1,1);      latency_cpu : REAL;      METHOD : COG;      ackcount_throughput : REAL;      DEFAULT:= 0;END_VAREND_DEFUZZIFYVAR_OUTPUTRULEBLOCK      result_anomaly : REAL;      AND : MIN;END_VAR      ACT : MIN;FUZZIFY:  latency_cpu      ACCU : MAX;      TERM low:=   (0,1)   (0.2,0);      RULE   1  :   IF  latency_cpu  IS  low      TERM medium:=   gauss    0.5    0.12;AND  ackcount_throughput  IS  low  THEN      TERM high:=   (0.75, 0)    (1,1);result_anomaly IS high;END_FUZZIFY      RULE   2  :   IF  latency_cpu  IS  highFUZZIFY:  ackcount_throughputOR  ackcount_throughput  IS  high  THEN      TERM low:=   (0,1)   (0.2,0);result_anomaly IS low;      TERM medium:=   gauss    0.5    0.12;      RULE   3  :   IF  latency_cpu  IS  medium      TERM high:=   (0.75, 0)    (1,1);AND ackcount_throughput IS medium THENEND_FUZZIFYresult_anomaly IS medium;DEFUZZIFY:  result_anomalyEND_RULEBLOCK      TERM low:=   (0,1)    (0.2,0);END_FUNCTION_BLOCK  

The FIS uses two fuzzy input variables (*i.e.*, the estimated probability on latency and CPU utility denoted by latency_cpu and the estimated probability on ack count and throughput denoted by ackcount_throughput) and one fuzzy output variable. The output variable shows how likely the anomaly is, given the two input fuzzy variables. Each fuzzy variable (including input and output variables) is associated with three fuzzy numbers to define “low”, “medium” and “high” estimated probabilities. To give a crisp FIS output indicating the anomaly likelihood, the FIS uses the Centre of Gravity (COG) method to de-fuzzify the fuzzy output, as shown in Algorithm 1.

The bottleneck detector detects a topology task’s performance degradation which can be caused by a CPU or a Java heap/non-heap overhead, an exception in the code, network traffic, and so forth.

### 3.4. Router

Efficiently utilizing network resources is critical to ensure real-time performance in big data environments. The router component is designed to process a tuple with a minimum network cost. Whether the router is triggered or not depends not only on the signals sent by the bottleneck detector, but also on the grouping specification devised between one component layer and its subsequent layer, which is affected by domain logics. The idea behind the router component is trying to make the messaging happen in the same worker process where possible. One thread (a task) only needs to send the “pointer” of a message to a thread (a target task) that resides in the same worker (a JVM). If there exists such a target task in the same worker, the router then looks for a target task that resides in the same node (a task that resides in a different slot, but in the same machine), which also tries to save network resources. If there exists no such target task, then the tuple is transferred to another node.

**Figure 6:**
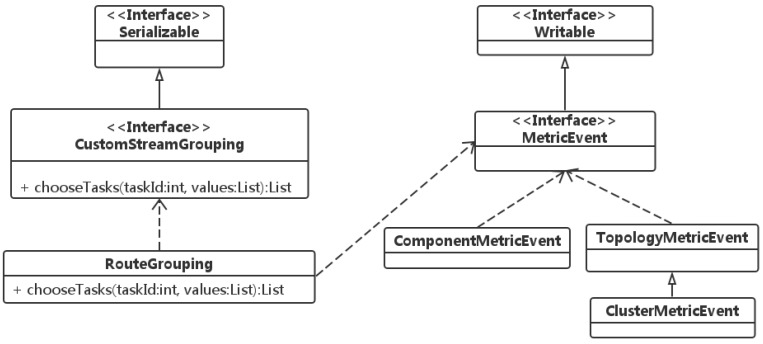
Core classes of tuple routing.

The core class diagram is shown in Figure 6. The routing scheduling is implemented from two parts of the work. First, a new grouping class RouteGrouping should implement Storm’s built-in grouping CustomStreamGrouping, which is used for extensions of the user-defined grouping mechanism. Second, the *chooseTasks* method provides the IDs of downstream bolts and the current tuples to be sent. According to the routing mechanism described above, in the *chooseTasks* implementation, the current states of a Storm cluster and the running topology should be available, which is the reason why RouteGrouping depends on MetricEvent.

The routing algorithm is implemented in *chooseTasks* and is detailed in Algorithm 2. The algorithm first finds the set of tasks that the tuple is going to be sent to by (note that the algorithm input can be obtained from Nimbus using an RPC protocol implementation Thrift and the information attached within the tuple):

$$TaskIDs=map3.get(map2.get\left(map1.get\left(taskID_{t}\right)\right)$$

Then, it iterates each task to find its location (to find which process/slot in which host for the task) by $\left(slotID_{i},hostID_{i}\right)$ = map4.get($taskID_{i})$ and to check if the host where the task is located is the same as the host where the tuple currently is located. If yes, then check if the two tasks (the currently iterated task and the task where the tuple currently is located) are in the same slot. Finally, the best task is found to route the tuple.

The default grouping shown in the algorithm refers to the shuffle grouping in Storm. It does not include other groupings, such as global grouping and field grouping, because these groupings are more restricted to business logics, which the router cannot violate. This means that the routing algorithm is only fit for the shuffle grouping, while other groupings (direct grouping, global grouping and field grouping) are not fit.

Nimbus uses a scheduler to assign tasks to supervisors. The default scheduler aims at allocating computing resources evenly to topologies. There are some works on scheduling topologies online. An example work proposes an online schedule that not only takes into account the component order, but also the run-time awareness, like stream groupings [3]. Although it economizes CPU and network resources, rescheduling (schedule a running topology) is a bad idea, especially for some cases, such as video object detection, target tracking and video scenario sensing. The reason is that a rescheduling will cost a relatively long time. It brings the video streams’ long waiting time for the redeployment, which affects performance severely. A rescheduling will also affect the performance of the bottleneck detector, because the metrics will show an abnormal pattern when rescheduling.

**Figure 7:**
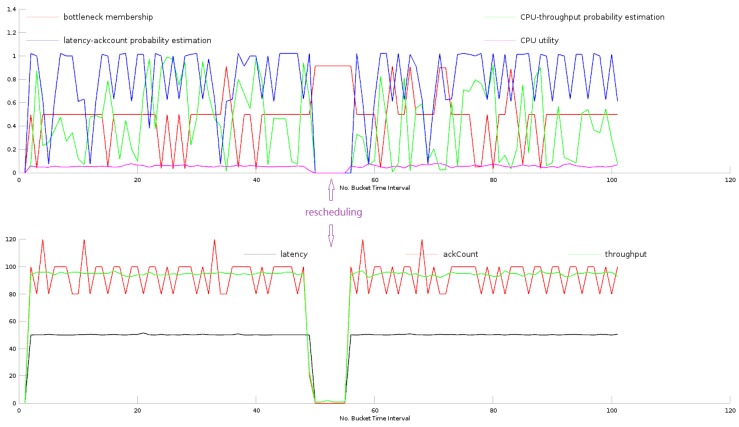
Metric patterns when rescheduling a topology.

Figure 7 shows the metric patterns during rescheduling a topology. It is not surprising that during rescheduling, the four tracked metrics all witness a sharp fall to zero while the bottleneck membership goes up to near one, indicating that the metric time series pattern seems to be a bottleneck, whereas it is not a bottleneck, but just a rescheduling action.


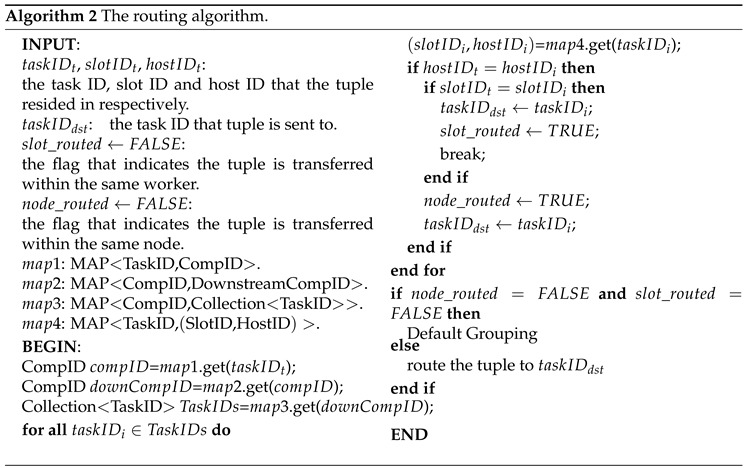


### 3.5. CPU-GPU Switcher

Modern GPUs are very efficient at manipulating computer graphics and image processing, and their highly parallel structure makes them more effective than general-purpose CPUs for algorithms where the processing of large blocks of data is done in parallel. Many algorithms of video and image processing can be paralleled on a GPU, such as MOG (Gaussian models for foreground subtraction). We provide an alternative set of OpenCV-based GPU APIs to ensure real-time performance, that is to ensure the required frame rate (frame per second). The proposed CPU-GPU switcher provides an alternative set of OpenCV-based GPU APIs to achieve performance accelerations. The Java OpenCV provides CPU interfaces based on which we implement a set of GPU interfaces without modifying the original APIs.

**Figure 8:**
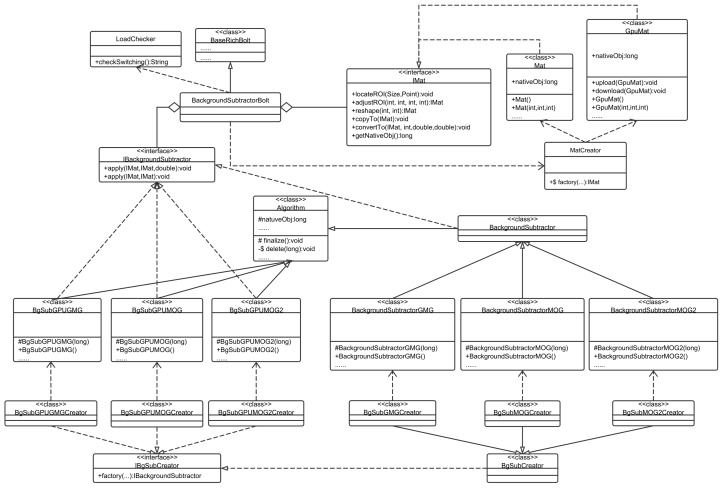
CPU-GPU switching architecture illustrated with background subtraction.

A typical architecture of CPU-GPU collaboration for video processing is the background subtraction, as shown in Figure 8. The switching algorithm is wrapped in the LoadChecker class. When the worker containing the BackgroundSubtractorBolt is assigned to a slot, it checks the node environment and instruct the MatCreator and the IBgSubCreator to choose APIs and the type of background subtraction algorithms.

From the perspective of resource maximum utilization, when a topology is submitted to Storm, we have to consider the available hardware resources. In general, if the CPU is the bottleneck and affects the real-time performance, but enough RAM is available, it is reasonable to switch the work to the GPU. Furthermore, from the perspective of QoS assurance, the required frame rate must be ensured in order to meet the business requirement. When a Storm cloud is housing an increasing number of topologies, it becomes essential the CPU and GPU to collaborate for both resource maximum utilization and QoS assurance.

The CPU-GPU switching algorithm is shown in Algorithm 3. The CPU-GPU switcher periodically (within time interval *T*) calculates the FPS (Frame Per Second) and compares it to the required frame rate (RFR). When the calculated FPS is detected to be less than the required frame rate, it switches workloads from the CPU to the GPU.

**Algorithm 3** CPU-GPU switching algorithm. **INPUT**: RFR: required frame rate. *T*: time interval for each frame rate computation. toGPU←FALSE: flag indicating if switching workloads to GPU. **BEGIN**: **for each** time interval *T*
**do**  compute the number of tuples *N* passing through the task within *T*;  $fps\leftarrow \frac{N}{T}$;  **if**
fps<RFR
**then**   toGPU←TRUE;  **end if**  **if**
toGPU==TRUE
**then**   Switch workloads to GPU;   **break**;  **end if** **end for**

## 4. Evaluation

In this section, we focus on the evaluations on the component performance. We first submit the target topology and then submit bottleneck-maker topologies one by one, which are categorized into two types. One is the CPU-intensive type of topology, and the other is the I/O-intensive type of topology.

We do not consider the memory-intensive type of topology, because each worker starts running with a predefined memory configuration. If we force the increase of the memory that one worker occupies, the worker will end up with an “out of memory” exception. However, in the proposed paper, we do not consider the program exception anomalies. We summarize the proposed components as discussed in the former section, as shown in Table 1.

The idea is that when the bottleneck detector gives alarms, the router will be triggered to save the network cost or the switcher will be triggered to ensure the required frame rate by utilizing the GPU. These components are evaluated from two threads, which are:

- *Thread*-1: evaluates the router and the bottleneck detector under the CPU-intensive and I/O-intensive topologies, respectively.
- *Thread*-2: evaluates the CPU-GPU switcher and the bottleneck detector under the CPU-intensive and I/O-intensive topologies, respectively.

In the following subsections, we first set up the evaluation environment, then show the evaluations in the two bottleneck cases as mentioned above.

### 4.1. Environment Configurations

Our testing environment is running on five computing nodes (supervisor) and one master node, each of which is configured in Table 2 and Table 3.

**Figure 9:**
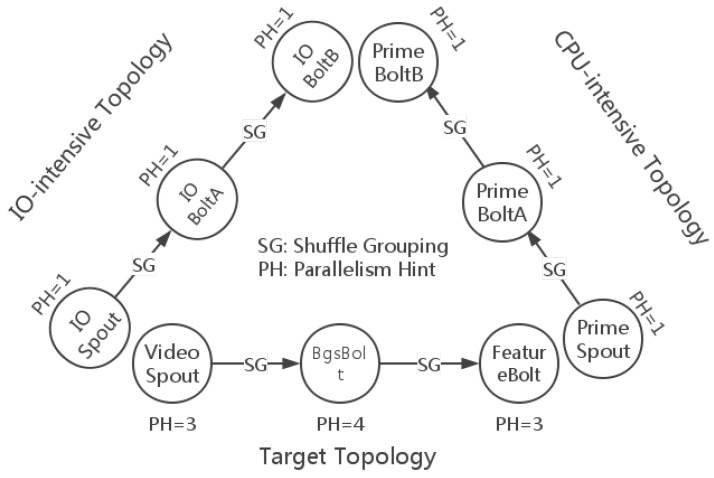
Topologies for evaluation.

The test topologies with grouping specifications and parallelism hints are illustrated in Figure 9. The target topology is the one that we want to evaluate, and the remaining two topologies are the bottleneck makers. VideoSpout is responsible for receiving video streams and decoding them into frames. BgsBolt subtracts the foregrounds of frame sequences and rectangles each moving object in the frame. FeatureBolt extracts the SURF features [8] of the moving objects. Finding out the maximum prime is considered as CPU-intensive work, because it involves many big integer divisions. The CPU-intensive topology finds the maximum prime in a range in each task. Additionally, the I/O-intensive topology transfers tuples in a very high frequency and does not do any application-level work.

### 4.2. Performance

As mentioned above, *Thread*-1 evaluates the router, and *Thread*-2 evaluates the switcher. In this section, we evaluate the two threads separately.

From Figure 9, we can see there are 10 tasks (three tasks for VideoSpout, four tasks for BgsBolt and three tasks for FeatureBolt) for the target topology. The Storm default scheduler uses a round-robin strategy to deploy bolts and spouts, so that each node in a topology has almost an equal number of component instances running in each slot, even for multiple topologies running in the same cluster.

To make it more clearer, we illustrate the mapping of the tasks of the target topology to the five computing nodes using the Storm default scheduler, which is shown in Table 4. According to Figure 9, which shows the deployment of the target topology, we focus on the four tasks of the BgsBolt for the reason that it is the most CPU-intensive bolt among the components in the topology and that it is located in the medium layer, which indicates that the whole traffic of the topology has to pass through the layer. For the two bottleneck makers, *i.e.*, the I/O-intensive topology and the CPU-intensive topology, the Storm default scheduler will also use the round-robin strategy to schedule each task that will be assigned to slots in the five nodes.

If the parallelism hint of a component named *X* is *N*, we denote the tasks/instances of the component by X−1, X−2, ..., X−N. For example, when an I/O-intensive topology is being submitted to the Storm cluster, since there is only one instance for each component (the parallelism hint is one), the Storm default scheduler might first assign the IOSpout-1 to Node 1, IOBoltA-1 to Node 2 and IOBoltB-1 to Node 3. After the above submission, if a new CPU-intensive topology is being submitted, then similarly, PrimeSpout-1 will be assigned to Node 4, PrimeBoltA-1 to Node 5 and PrimeBoltB-1 to Node 1.

#### 4.2.1. Thread-1 Results

**Figure 10:**
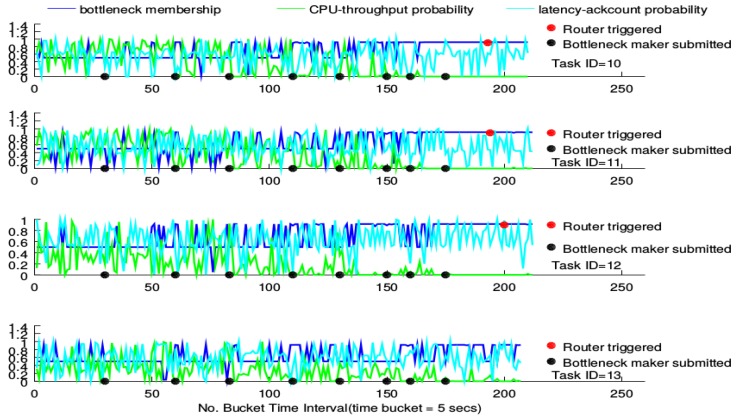
Router performance under the CPU-intensive bottleneck maker.

**Figure 11:**
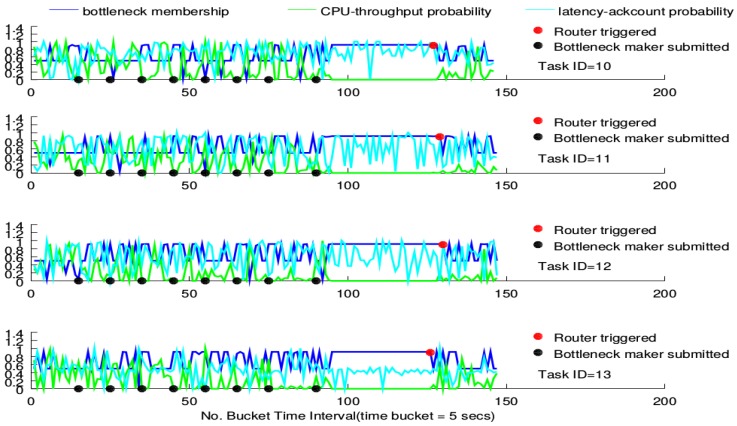
Router performance under the I/O-intensive bottleneck maker.

The router performance on the four tasks of the BgsBolt is shown in Figure 10 and Figure 11. Figure 10 illustrates how the router performs under CPU-intensive bottleneck makers (also called topologies) whose configurations are detailed in Figure 9. Similarly, Figure 11 illustrates how the router performs under I/O-intensive bottleneck makers.

For each figure, there are four sub-figures, each of which shows how the router performs under a BgsBolt task. For each sub-figure, there are three curves. At each time-stamp, values of four metrics (CPU utility, throughput, latency and ack count) of the BgsBolt task are sampled and fed into the two joint probability distributions (the CPU-throughput joint distribution and latency-ack count joint distribution). For one new metric from Storm, each joint distribution will generate a probability estimation. Generally, the two generated estimations indicate how frequent the sampled metrics occur under historical metrics and are shown in the sub-figure (in cyan and green). The two generated estimations are then regarded as the input of the FIS detailed in Algorithm 1, which produces a crisp membership value (shown in blue) indicating the likelihood that the two generated estimations are from the task with the bottleneck. Therefore, for each time-stamp, there are three points (two generated estimations in cyan and green and one crisp membership value in blue). The evaluation idea is to check how the three points behave under the circumstance of increasing numbers of bottleneck makers (the time of each bottleneck maker’s submission is doted in black) along with time. In order to test the performance of the router, we give the definition of the bottleneck for *Thread*-1 as:

**Definition 1** (Task bottleneck).  A task has a performance bottleneck when the FIS output (*i.e.*, the bottleneck membership) stays larger than 0.9 for the past 30 time buckets.

Before a bottleneck occurs, the router will not be triggered. The given bottleneck is treated as an indicator to trigger the router; therefore, checking the performance of the router mainly referring to the three points’ patterns along with time before and after the router gets triggered.

The triggering of the router is represented as a red point in the sub-figure. For each task, the bottleneck membership that exceeds 0.9 turns out more frequently along with the increasing number of submitted bottleneck makers. Furthermore, the CPU-throughput probability estimation gradually declines while the latency-ack count probability estimation does not witness any declining trends. For the CPU-intensive case, all routers, except for the one devised on Task 13, are triggered around the 200th time-stamp. For the I/O-intensive case, each task’s router is triggered around the 135th time-stamp. This conforms to the measurement in Definition 1. Furthermore, no significant declining in the membership is witnessed after the triggering; while for the I/O-intensive case, however, low bottleneck membership occurs, and the CPU-throughput probability goes up.

#### 4.2.2. Thread-2 Results

**Figure 12:**
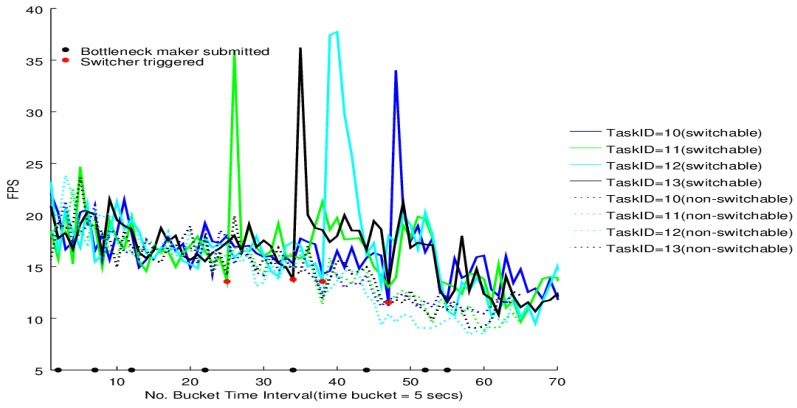
Switcher performance under the CPU-intensive bottleneck maker.

**Figure 13:**
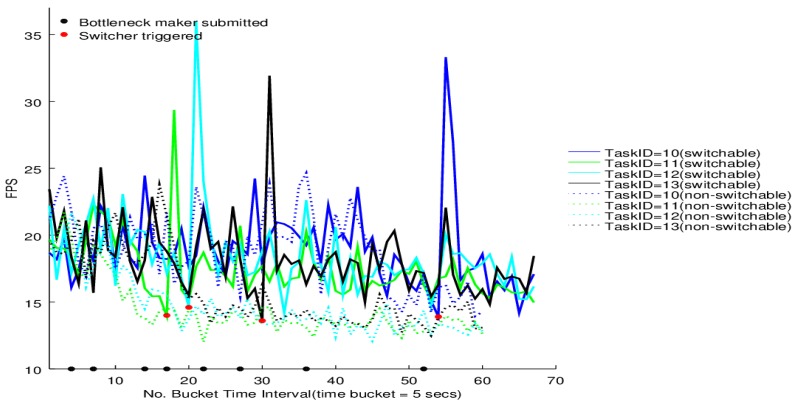
Switcher performance under the I/O-intensive bottleneck maker.

The switcher performance on the four tasks of BgsBolt under CPU-intensive and I/O-intensive bottleneck makers is shown in Figure 12 and Figure 13, respectively. Each BgsBolt task has an ID. In this evaluation, the four corresponding IDs for the four BgsBolt tasks are 10, 11, 12 and 13, as shown in the two figures. Each task (illustrated with a different colour) is evaluated with a switcher (illustrated with full lines) and without a switcher (illustrated with dashed lines). The switcher is triggered when the bottleneck occurs. We define the bottleneck for *Thread*-2 as:

**Definition 2** (Task Bottleneck).  A task has a performance bottleneck when the average FPS within a single time bucket interval is less than 14 (the required frame rate).

If we set the sampling time interval to 5 s, then the *RFR* = 14 and *T* = 5 *s*, which are the inputs in Algorithm 3. Along with the increasing number of bottleneck makers, an overall decline in FPS for both the CPU-intensive case and I/O-intensive case is illustrated. Each task witnesses a jump upon the switcher being triggered (the bottleneck occurs) and then falls down to the original level. Furthermore, for the non-switchable case, no switcher can be triggered when the FPS is lower than the required frame rate. Therefore, it is not surprising to see that the switchable case outperforms the non-switchable case after triggering.

### 4.3. Discussion

The results show that the proposed framework can help achieve higher dependability for Storm-based stream processing. When the throughput decreases to a certain degree, the resource switcher will start the GPU processor, ending up with a recover in the throughput. There are, however, some times right after the switching when the throughput witnesses bumping-up. This is because before putting the GPU into practical operations, the CUDA setup costs some time that is necessary to initialize the CUDA context on the GPU, allocate memory and release the CUDA context. After the initialization, the context is kept alive to avoid the overhead in every new CUDA computation. The bump-up occurs in that, during the context initialization, sequences of video frames were being appended in the receiving message queue of the video task, which was withstanding increased appending pressure. After the context is kept alive and ready to run, the pended sequences of video frames are pulled into the GPU, whose high performance makes the throughput bump up and consumes the frames in the receiving message queue, ending up with a fall after the bump-up.

Furthermore, it is interesting that there is a strong standard deviation in the FPS patterns along with time for the evaluation results. FPS computation can also be interpreted as $\frac{1}{\delta T_{t\to t+1}}$ where $\delta T_{t\to t+1}$ is the time difference of two adjacent tuples processed in one task. The strong standard deviation exists because all tasks of the target topology are distributed across the Storm cloud (as shown in Table 4), which means each tuple generated from the three VideoSpout instances has to go through the down-stream component instances. Unlike some standalone video applications, which usually show nearly constant timeliness and load characteristics (for example, playing a movie via a video player on a laptop where the timeliness requirements are met and there is a high likelihood that the load is constant over time), with the increasing number of bottleneck-maker topologies that are submitted to the Storm cloud, hundreds of thousands of tuples are passing across the network, which will cause a time delay for some tuples, while some others not. Furthermore, object serialization/deserialization is unavoidable when an object is sent from a node to another in a distributed environment. Another important factor contributing to the strong standard deviation is the serialization/deserialization of relatively big video frames compared to text data. Therefore, the $\delta T_{t\to t+1}$ can be very deviated for two different tuples, which causes the strong standard deviation in the FPS patterns.

**Figure 14:**
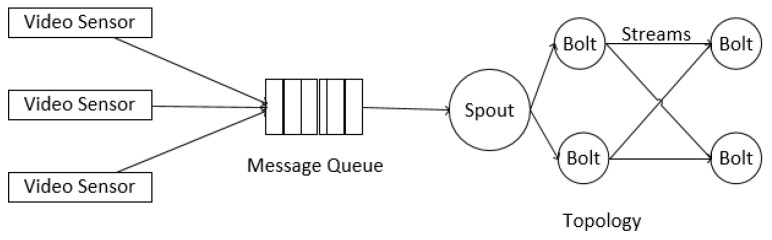
A general video processing system using Storm.

Usually, video analytic applications require that the Sampling Frame Rate (SFR) is constant/low deviation; because high deviation cannot ensure video analytic algorithms with temporal characteristics to be effective. For example, using particle/Kalman filters to track video objects requires SFR to be constant, which is also the same with video object detection algorithms, such as Gaussian mixtures and frame difference. However, will the high deviation in Figure 12 and Figure 13 affect the performance of video analytic algorithms with temporal characteristics? Actually, it will not, because the high deviation in Figure 12 and Figure 13 is not really the high deviation in SFR. Look at the architecture of a general data processing platform with Storm in Figure 14. The SFR corresponds to the frames sampled at the video sensor, while the Processing Frame Rate (PFR) corresponds to the frames processed in bolts. Patterns in SFR and PFR in a system can be very different. What really matters for an algorithm’s performance is not PFR, but SFR. If SFR has high deviation, then algorithms can be very poorly efficient. However, if PFR has high deviation, but SFR is constant, algorithms with temporal characteristics can also ensure their performance, in which case, the only requirement is that PFR should be larger than SFR (frames processed per second larger than sampled per second); otherwise, the message queue will be overloaded. Therefore, the high deviation in PFR has neglectable influence on the applicability of Storm-based video processing. We have other experiments to show the applicability, as in [9,10].

Previously, we described the router performance under the increasing number of CPU-intensive and I/O-intensive bottleneck makers, respectively, shown in Figure 10 and Figure 11. For both the CPU-intensive case and the I/O-intensive case, they all show the common patterns that, along with the increasing number of topologies submitted to Storm, the probability estimations show a very different pattern. This means that the new estimations are very different from their historical estimations, which results from the resource bottleneck caused by the bottleneck makers’ continuous submission to Storm. Although for both cases, the bottleneck membership shows a similar pattern according to the logic rules in the fuzzy inference system before the triggering, it shows a significantly diverse pattern after the triggering for the CPU-intensive and I/O-intensive cases. The bottleneck membership declines for the I/O-intensive case, while it does not change for the CPU-intensive case, which means that the trigger proposed in this paper works for the network bottleneck, but does not really work for the computing bottleneck. This is rooted in that the routing algorithm only considers the configuration of tasks’ mapping into the physical computing nodes and guides the tuples’ mapping among tasks. It does not consider the computing resources. Although the bottleneck does occur in both cases, it results from different sources. For the CPU-intensive case, the bottleneck results from a lack of computing resources. For the I/O-intensive case, the bottleneck results from the lack of network resources. Therefore, the router can have a good performance when the bottleneck results from a busy networking communication in Storm, but fails to work when the bottleneck is rooted in a lack of computing sources.

## 5. Related Work

There are some works on CPU-GPU collaborations. Takizawa *et al.* [11] present a runtime environment SPRAT(Stream Programming with Runtime Auto-Tuning) that dynamically selects an appropriate processor, so as to improve the energy efficiency. Breß *et al.* [12] propose heuristics that optimize database query processing for response time and throughput based on load-aware inter-co-processor parallelism. Furthermore, Lang *et al.* [13] develop an energy-aware model to predict how to distribute the data to the processing units to achieve the most energy-efficient execution. The above efforts are mainly focused on a CPU-GPU-based collaboration computing; in this paper, we also propose a similar processor switching method to maximize the computer resources. The difference is that they do not focus on a real-time stream processing environment.

Some efforts are devoted to improve Storm-based applications. Xia *et al.* [14] propose to put a cloud-based web server on Storm. Tseng *et al.* [15] use Storm to analyse data from a large number of GPS vehicle probes, and Kumar *et al.* [16] use Storm to find out the anomalies in a Water Distribution Network (WDN) topology. We can borrow some ideas on optimizing the usage of Storm, but none of these work focus on improving the dependability of the Storm-based stream processing.

Another part of the related work is the analysis and optimization of Storm. The network bandwidth is a vital bottleneck for real-time distributed frameworks. Lutz *et al.* [2] presents NaaStorm, which is an implementation of Storm modelled on NaaS (Network as a Service). It provides top-level support for controlling the locality of computations in Storm, thus making the network infrastructure more transparent to tenants. Based on a round-robin mechanism, the default scheduler of Storm evenly distributes the execution of topology components on available machines, yet disregards the network cost when tuples go through the whole topology. Aniello *et al.* [3] propose a new schedule (*i.e.*, assignment of tasks to machines) for Storm to monitor system performance and reschedule the deployment at run-time. Fischer *et al.* [4] and Xu *et al.* [5] also propose new schedules that can detect network traffic and reschedule a topology execution. Ghaderi *et al.* [17] and Fischer *et al.* [18] investigate the problem of scheduling/partitioning graphs on Storm to satisfy load balancing and cost considerations. All of these methods improve the performance compared to Storm’s default scheduling. These works give us hints on optimizing Storm. However, apparently, we have different targets, which focus on the real-time big video processing.

This existing worker-level granularity scheduling means coarsely migrating processes among computing nodes, which is too heavy weight for real-time video processing. Even though only one worker needs scheduling, the whole topology containing the worker must stop and restart, which costs 5 to 30 s in our experiments. It is unacceptable for some time-critical applications. Instead of scheduling topologies, the proposed router schedules tuples from a fine-grained level without consuming the time of scheduling topologies. Furthermore, the existing work on scheduling Storm considered only the network and, yet, not computing resources. However, the scheduling of computing resources is important in the context of real-time analysis of big video streams, and the proposed framework uses the CPU-GPU switcher to build up a collaboration of heterogeneous processors.

## 6. Conclusions and Future Work

Improving the dependability of the Storm platform is important for usable stream processing. The existing works’ scheduling granularity is too coarse to have good performance, which is designed at a Storm worker level that migrates processes among computing nodes. Additionally, the existing work on scheduling Storm considered only the network, but computing resources should be considered, as well. In this paper, we propose Healthcare4Storm, a framework that finds Storm insights based on various metrics, builds up knowledge on its running status and finally ends up with smart scheduling decisions. It takes both network and computing resources into account and, from a fine-grained level, schedules tuples instead of topologies. The evaluations show that Healthcare4Storm has good potentials to improve the capabilities of Storm and to enhance the dependencies of stream processing applications.

There are several aspects that need improvements. First, the proposed framework will be extended to take memory consumption besides network and computing resources into account, to make better decisions for the video processing tasks in Storm. Second, there are many anomaly detection techniques instead of the fuzzy inference system, such as deep learning models, Bayesian models and other time series models. We are planning to explore these anomaly detection techniques to automatically detect anomalies and to compare the performance.

## Figures and Tables

**Table 1:** Component functionality summarization.

Component	Functionality
Metric Sensor	c devised on a monitored topology from which metrics are collected, cleaned and formatted.
Bottleneck Detector	analyses the metrics and gives alarms when bottleneck occurs.
Router	triggered to save the cloud’s network cost upon bottleneck occurrence.
CPU-GPU Switcher	triggered to ensure the required frame rate upon bottleneck occurrence.

**Table 2:** CPU and GPU parameters for a computing node.

Device	Total Memory	Number	Clock Speed
GeForce GTX 745	4 G (Graphics Memory)	384 CUDA Cores	1.03 GHz
Intel core i7-4790	8 G (RAM)	8 Cores	3.60 GHz

**Table 3:** Configurations.

	Item	Value
Software	Storm	0.9.3
	ZooKeeper	3.3.5
	OpenCV	2.9.0
	JDK	OpenJDK 7
	Hadoop	2.5
Hardware	OS	Ubuntu Linux Version 2.6
	CPU	Intel core i7-4790 @ 3.60 GHz
	RAM	8.00 GB
	SCSI	20.00 GB

**Table 4:** Mapping of the target topology’s tasks to the five computing nodes.

	Node 1	Node 2	Node 3	Node 4	Node 5
Tasks	VideoSpout-1	VideoSpout-2	VideoSpout-3	BgsBolt-1	BgsBolt-2
	BgsBolt-3	BgsBolt-4	FeatureBolt-1	FeatureBolt-2	FeatureBolt-3

## References

[1] Huang T. Surveillance Video: The Biggest Big Data. https://www.computer.org/web/computingnow/archive/february2014.

[2] Lutz C. (2012). Enhancing the Performance of Twitter Storm With in-Network Processing. Bachelor’s Thesis.

[3] Aniello L., Baldoni R., Querzoni L. Adaptive online scheduling in storm. Proceedings of the 7th ACM International Conference on Distributed Event-Based Systems.

[4] Fischer L., Scharrenbach T., Bernstein A. Network-aware workload scheduling for scalable linked data stream processing. Proceedings of the International Semantic Web Conference (Posters & Demos).

[5] Xu J., Chen Z., Tang J., Su S. (2014). T-storm: Traffic-aware online scheduling in storm. Proceedings of the 34th IEEE International Conference on Distributed Computing Systems (ICDCS).

[6] Jouffe L. (1998). Fuzzy inference system learning by reinforcement methods. IEEE Trans. Syst. Man Cybern. Part C (Appl. Rev.).

[7] Bryan L.A., Bryan E.A. (1997). Industrial txt and videocompany. Programmable Controllers.

[8] Bay H., Ess A., Tuytelaars T., Gool L.V. (2008). Speeded-up robust features (surf). Comput. Vis. Image Underst..

[9] Zhang W., Xu L., Li Z., Lu Q., Liu Y. (2016). A deep-intelligence framework for online video processing. IEEE Softw..

[10] Zhang W., Xu L., Duan P., Gong W., Lu Q., Yang S. (2015). A video cloud platform combing online and offline cloud computing technologies. Pers. Ubiquitous Comput..

[11] Takizawa H., Sato K., Kobayashi H. (2008). Sprat: Runtime processor selection for energy-aware computing. Proceedings of the 2008 IEEE International Conference on Cluster Computing.

[12] Breß S., Siegmund N., Heimel M., Saecker M., Lauer T., Bellatreche L., Saake G. (2014). Load-aware inter-co-processor parallelism in database query processing. Data Knowl. Eng..

[13] Lang J., Rünger G. (2014). An execution time and energy model for an energy-aware execution of a conjugate gradient method with CPU/GPU collaboration. J. Parallel Distrib. Comput..

[14] Xia X., Tian L. (2014). A web server cluster solution based on twitter storm. J. Data Anal. Inf. Process..

[15] Tseng P.J., Hung C.C., Chuang Y.H., Kao K., Chen W.H., Chiang C.Y. (2014). Scaling the real-time traffic sensing with gps equipped probe vehicles. Proceedings of the 2014 IEEE 79th Vehicular Technology Conference (VTC Spring).

[16] Kumar S. (2014). Real Time Data Analysis for Water Distribution Network Using Storm. Master’s Thesis.

[17] Ghaderi J., Shakkottai S., Srikant R. Scheduling storms and streams in the cloud. Proceedings of the 2015 ACM SIGMETRICS International Conference on Measurement and Modeling of Computer Systems.

[18] Fischer L., Scharrenbach T., Bernstein A. Scalable linked data stream processing via network-aware workload scheduling. Proceedings of the 9th International Conference on Scalable Semantic Web Knowledge Base Systems.

